# Long COVID-19 Syndrome: Insights From a Major Tertiary Center in the UK on Who Is at Greater Risk

**DOI:** 10.7759/cureus.50027

**Published:** 2023-12-06

**Authors:** Saad Tariq Khan, Khalid Rashid, Farrukh Ansar, Muhammad Y Khan, Fahd Ali Khan, Rawan Ebrahim Husain Ahmed Ali Ismaeel, Raheem Hanif Mohammed, Rehan Mustafa, Bisma Tariq Khan, Behram Tariq, Mubbashar Husssain, Aamir Waheed

**Affiliations:** 1 Medicine, Darlington Memorial Hospital, Darlington, GBR; 2 General Internal Medicine, James Cook University Hospital, Middlesbrough, GBR; 3 Medicine, Northwest School of Medicine, Khyber Medical University, Peshawar, PAK; 4 Medical Education, Pak International Medical College, Peshawar, PAK; 5 Medicine, James Cook University Hospital, Middlesbrough, GBR; 6 Medicine, Shaukat Khanum Memorial Hospital and Research Centre, Lahore, PAK; 7 Internal Medicine, Northampton General Hospital, Northampton, GBR

**Keywords:** predictors of post covid-19 syndrome, post-covid-19 symptoms, covid-19 vaccine, covid-19, long covid-19

## Abstract

Introduction: The COVID-19 pandemic triggered the unprecedented 'long COVID' crisis, with persistent symptoms beyond two months post-infection. This study explores the nexus between long COVID symptoms, patient demographics such as age, gender, and smoking, and clinical factors like vaccination, disease severity, and comorbidities.

Methods: A retrospective analysis of records was conducted between September 2021 and December 2022. The analysis covered adults with confirmed COVID-19 diagnoses. Data encompassed demographics, medical history, vaccination, disease severity, hospitalization, treatments, and post-COVID symptoms, analyzed using logistic regression.

Results: Among 289 participants, the average age was 51.51 years. Around 62.6% were females, and 93% received the COVID-19 vaccination, i.e., primarily the mRNA vaccine (48.4%) and the adenovirus vector-based vaccine (34.8%). Reinfections occurred in 11.76% of cases. Disease severity varied, with 75% having mild, 15% having moderate, and 10% having severe infections. Hospitalization rates were significant (25.6%), including 10.7% requiring intensive care. Thirteen distinct post-COVID symptoms were reported. Fatigue, shortness of breath upon exertion, and brain fog emerged as the most prevalent symptoms. Notably, females exhibited higher symptom prevalence. Significant correlations were established between higher BMI and smoking with augmented symptomatology. Conversely, a link between booster doses and symptom reduction was discerned. Using multinomial regression analysis, gender and smoking were identified as predictors of post-COVID-19 symptoms.

Conclusion: The study underscores obesity, smoking, and the female gender's impact on long COVID symptoms; boosters show promise in alleviation. Respiratory pathology might underlie persistent symptoms in cases with radiological abnormalities and abnormal spirometry. Findings contribute to risk stratification, intervention strategies, and further research.

## Introduction

The COVID-19 pandemic has left an unprecedented mark on global health. The first reported case was in Wuhan, China, in December 2019 [1]. However, within a matter of months, it spread across the world to become a global pandemic [2]. As of August 2023, according to the WHO's COVID-19 dashboard, the total number of reported cases is more than 769 million, with over 6.9 million COVID-19-related deaths [3]. The COVID-19 pandemic has led to an increase in research aimed at understanding the complex factors that impact the progression of the disease and its long-term consequences.

Amidst the recovery phase, a novel phenomenon has emerged: long COVID syndrome. This condition is characterized by persistent symptoms in individuals who have apparently recovered from the acute phase of the COVID-19 infection. However, there is a lack of consensus regarding the exact definition of the term [4]. According to the WHO, long COVID is defined as "the continuation or development of new symptoms 3 months after the initial SARS-CoV-2 infection, with these symptoms lasting for at least 2 months with no other explanation" [5]. Different terminologies have been used to describe this condition, such as *post-acute COVID-19 syndrome*, *post-COVID-19 syndrome*, *post-acute sequelae of COVID-19*, and *post-COVID-19 condition* [6]. We use the term *long COVID* in this study.

The prevalence of long COVID symptoms varies in studies, ranging from 4.7% to 80% of patients [7]. In non-hospitalized patients, the prevalence of long COVID symptoms was found to be 35%. However, it was found to be around 87% in hospitalized patients [8]. One research study from Wuhan, China, showed that 76% of infected patients still experienced at least one symptom after being discharged from the hospital [9].

A wide spectrum of symptoms, involving multiple systems, have been attributed to long COVID syndrome. These symptoms include fatigue, breathlessness, brain fog, myalgias, anxiety, and depression [10]. The term ‘brain fog’ has been used by many patients to describe their lack of concentration, feelings of confusion, and having fuzzy thoughts [11]. Dyspnea has been reported as one of the most common symptoms in long COVID patients. Studies have shown that 5% to 81% of patients who were hospitalized during acute illness experienced persistent dyspnea even after being discharged. On the other hand, the prevalence of persistent dyspnea in non-hospitalized patients was around 14% [12]. The extensive symptom profile in patients with long COVID may indicate that it is a multi-factorial phenomenon with a complex pathophysiology.

The pathophysiology of long COVID is still unclear. However, several possible mechanisms have been suggested to explain the aetiology of long COVID. One such proposed mechanism includes immune-related microvascular inflammation and thrombosis. One study pointed out that there were few changes in CD8+ T cells lasting up to six months following hospital discharge [13]. These changes may be responsible for a persistent inflammatory response, which contributes to the incidence of long COVID. The neurocognitive symptoms of long COVID syndrome, such as brain fog, fatigue, anosmia, and anxiety, have been associated with elevated levels of anti-nuclear antibody (ANA) titers [14]. Significant changes have been found in tryptophan metabolism in COVID-19-recovered patients [15]. Tryptophan is a precursor of melatonin and serotonin, which play vital roles in controlling sleep and mood disorders. Additionally, tryptophan is involved in skeletal muscle regulation. This could explain the sleep and mood disorders, as well as unexplained features such as myalgia in long COVID.

As COVID-19 is a recent phenomenon, there is a paucity of research regarding the various risk factors and predictors of long COVID. As it gains more recognition, there is a growing interest in understanding the potential association between long COVID and different demographic and clinical factors. The present study aims to uncover potential correlations between long COVID symptoms and various patient characteristics, including age, sex, smoking habits, vaccination status, severity of the disease during the acute phase, and the presence of co-existing health conditions. This research makes a valuable contribution to the expanding knowledge base that informs clinical management, public health strategies, and future research efforts in understanding the long-term consequences of the COVID-19 pandemic.

## Materials and methods

This retrospective study was performed at the South Tees NHS Foundation Hospital, United Kingdom, from September 2021 to December 2022. Prior to commencing the study, our team obtained ethical approval from the Health Research Authority and the South Tees NHS Foundation Hospital’s Ethics Committee. After the approval, we collected the data from the hospital database using the inclusion and exclusion criteria. Individuals aged 18 and above with a confirmed COVID-19 diagnosis through reverse transcription polymerase chain reaction (RT-PCR) testing and patients undergoing follow-up at the long COVID-19 clinic from September 2021 to December 2022 were included in the study. Patients below 18 years of age and those with incomplete or absent medical records were excluded. To gather the data, a well-structured questionnaire was devised. The questionnaire encompassed patient demographic particulars, concurrent medical conditions, vaccination history, including booster doses, initial COVID-19 infection year, potential disease recurrences, need for hospitalization, treatment administered, and post-COVID symptoms.

Furthermore, the severity of COVID-19 pneumonia was assessed, and patients were classified into three categories: mild, moderate, and severe. Patients who did not require hospital admission for COVID-19-related symptoms were classified as mild. Those who required hospital admission but did not receive invasive or noninvasive ventilation or any other organ support and were not admitted to the critical care unit or high-dependency unit were deemed moderate. Patients who received mechanical or noninvasive ventilation or were admitted to critical care or the high-dependency unit for other organ support were considered to be severe.

To ensure the questionnaire's appropriateness and internal consistency, a pilot study was conducted using 20 patient record files. The findings from this pilot study helped identify any areas for improvement. Additionally, the questionnaire underwent review by three independent consultants, who provided valuable feedback that was incorporated to enhance the quality of data collection. The data extraction process involved a comprehensive review of the patient's record files, and the information was meticulously documented in a printed questionnaire format. Subsequently, the gathered responses were imported into SPSS Statistics version 23.0 (IBM Corp., Armonk, NY, USA). The variables were appropriately adjusted based on their numeric and string properties to facilitate the most suitable statistical analysis.

To summarize the collected data, descriptive statistics, such as frequencies, means, and standard deviations, were calculated. For the statistical analyses, several methods were employed, including Pearson's chi-squared and correlation tests, Fisher’s exact test, and multivariate logistic regression analysis. In addition, odds ratios (ORs) and their corresponding 95% confidence intervals (CIs) were estimated to assess the associations between different variables. The statistical significance was determined by a two-sided p-value of less than 0.05, indicating a meaningful relationship or effect.

## Results

In this study, a total of 289 patients were included as participants. The mean age of the patients was determined to be 51.51 ± 13.14 years, with an age range spanning from 18 to 89 years. Among the participants, there were 108 male patients and 181 female patients, indicating a higher representation of females in the study population. The mean BMI of the patients was 27.43 ± 2.91 (range: 22 to 40). Regarding the ethnic composition of the sample, the majority of patients identified as British (n = 264). Additionally, there were 25 patients from various other ethnic groups included in the study. The smoking habits of the patients revealed that 207 individuals were non-smokers, indicating a considerable proportion of the sample population. Furthermore, 54 patients reported being ex-smokers, while 28 individuals identified themselves as active smokers. In terms of alcohol consumption, the majority of patients (n = 285) reported being regular alcohol consumers. However, a small subset of the population consisted of non-alcoholic consumers (n = 4). Regarding the COVID-19 vaccination status, a significant majority of the patients (93%) had received vaccination against the virus. The most administered vaccine among the vaccinated individuals was the mRNA vaccine (48.4%), followed by the adenovirus vector-based vaccine (34.8%). This highlights a trend of high vaccination rates within the study population. Lastly, it is noteworthy that a subset of patients (n = 34, 11.76%) experienced more than one occurrence of COVID-19 infection during the study, indicating the presence of reinfections within the cohort. Baseline characteristics of the study population are shown in Table 1.

**Table 1:** Baseline characteristics of the study population

Variable	N = 289 (%)
Gender N (%)	
Male	108 (37.37)
Female	181 (62.63)
Ethnicity N (%)	
British	264 (91.35)
Non-British	25 (8.65)
Smoking Habits N (%)	
Smoker	28 (9.68)
Ex-smoker	54 (18.68)
Non-smoker	207 (71.62)
Alcohol Habit N (%)	
Consumers	285 (98.61)
Non-consumers	4 (1.39)
Vaccination status N (%)	
Vaccinated for COVID-19	269 (93.07)
Non-vaccinated for COVID-19	20 (6.92)
Booster dose availed	240 (83.04)
Booster dose availed	49 (16.95)
Co-morbidities N (%)	
Hypertension	65 (22.49)
Diabetes mellitus type 2	40 (13.84)
Ischemic heart disease	20 (6.92)
Chronic kidney disease	18 (6.22)
Asthma	64 (22.14)
Immunosuppressive medicine	10 (3.4%)
Severity of COVID-19 Infection N (%)	
Mild	217 (75.1%)
Moderate	43 (14.9%)
Severe	29 (10%)

Among the patients included in our study, the severity of COVID-19 infections was also calculated, and it was revealed that 75% of the patients had mild disease, followed by 15% with moderate disease and 10% with severe infection. A notable proportion, specifically 78 individuals (25.6%), required hospital admission following their COVID-19 infection. This emphasizes the severity of the disease in a substantial subset of the study population. Within the hospitalized group, a subset of patients, comprising 31 individuals (10.7%), necessitated ICU care, indicating the critical condition of their illness. Additionally, 12 patients (4.63%) required intubation during their hospital stay, indicating the need for mechanical ventilation to support their respiratory function. During their hospitalization, various treatments were administered to manage the patients' conditions effectively. Among the patients, 68 individuals (23.52%) received antibiotics. Furthermore, 57 patients (19.72%) were treated with dexamethasone, a potent corticosteroid known for its anti-inflammatory properties in managing severe cases of COVID-19. In a smaller proportion of cases, specific therapeutics were employed. Nine patients (3.1%) received tocilizumab, a monoclonal antibody targeting the interleukin-6 receptor, which has shown efficacy in managing cytokine release syndrome associated with severe COVID-19. Additionally, four patients (1.4%) received remdesivir, an antiviral medication approved for the treatment of COVID-19, demonstrating its limited use within the study population. We ascertained a total of 13 distinct post-COVID-19 symptoms. Prevalent among these symptoms was fatigue, which manifested in approximately 90% of the cases. Subsequently, shortness of breath upon exertion was reported in 78% of the cases, followed by brain fog at 47%. Conversely, the symptom with the lowest incidence was the loss of libido, reported in a mere 1% of the cases. For a more comprehensive breakdown of symptom distribution, refer to Table 2.

**Table 2:** Distribution of post-COVID-19 symptoms in the study population GI: Gastrointestinal

Symptoms	Frequency (N = 289)	Male N (%)	Female N (%)	Smoker N (%)	Non-Smoker N (%)	Booster N (%)	No booster N (%)	Up to age 40 N (%)	Age 41-65 N (%)	Age 66-80 N (%)	Age over 80 N (%)
Fatigue	261 (90)	94 (87)	168 (92)	25 (89)	187 (90)	216 (90)	46 (94)	52 (58)	176 (92)	30 (88)	4 (66)
Myalgia	100 (34)	33 (30)	68 (38)	14 (50)	70 (33)	86 (36)	15 (30)	23 (40)	63 (32)	14 (41)	1 (16)
Brain fog	135 (46)	39 (36)	97 (54)	12 (43)	100 (48)	112 (46)	24 (49)	29 (50)	93 (49)	14 (41)	0 (0)
Shortness of breath	225 (77)	80 (74)	146 (81)	22 (79)	155 (75)	190 (79)	36 (73)	41 (70)	152 (80)	28 (82)	5 (83)
Anxiety	159 (55)	50 (46)	110 (60)	12 (43)	113 (55)	125 (52)	35 (71)	35 (60)	106 (55)	17 (50)	2 (33)
Anosmia	57 (19)	18 (16)	40 (22)	8 (29)	39 (19)	43 (18)	15 (30)	11 (19)	39 (20)	7 (20)	1 (16)
Hair loss	13 (4)	4 (3)	10 (6%)	1 (3)	12 (5)	13 (5)	1 (2)	2 (3)	10 (5)	2 (5)	0 (0)
GI disturbance	21 (7)	8 (7%)	14 (8%)	1 (3)	18 (9)	14 (6)	8 (16)	5 (8)	15 (8)	1 (3)	1 (16)
Migraine	34 (11)	6 (5%)	28 (15)	4 (14)	24 (12)	24 (10)	10 (20)	8 (13)	25 (13)	1 (3)	0 (0)
Loss of appetite	29 (10)	10 (9)	19 (10)	7 (25)	17 (8)	21 (9)	8 (16)	6 (10)	19 (10)	3 (8)	1 (16)
Loss of libido	3 (1)	2 (2)	1 (0.5)	1 (3)	2 (1)	2 (0.8)	1 (2)	0 (0)	3 (1.5)	0 (0)	0 (0)
Persistent body aches	78 (27)	25 (23)	53 (29)	9 (32)	56 (27)	60 (25)	18 (37)	13 (22)	55 (29)	10 (30)	0 (0)
Recurrent respiratory tract infections	25 (9)	10 (9)	15 (8)	5 (17)	16 (8)	20 (8)	5 (10)	3 (5)	17 (9)	4 (11)	1 (16)

The current study sheds light on the intricate manifestation of these symptoms within distinct groups. Gender-wise, an average of 3.50 ± 2.08 symptoms were observed in males, contrasting with a mean of 4.24 ± 1.99 symptoms in females. Stratified by age, the mean number of symptoms displayed variability: 3.93 ± 1.84 in those up to 40 years, 4.04 ± 2.15 in the age bracket of 41 to 65 years, 3.85 ± 1.82 among individuals aged 66 to 80 years, and notably lower at 2.66 ± 2.25 in those over 80 years. The distribution of co-morbidities further elucidated the spectrum of symptomatology, with 51.6% exhibiting no co-morbidities, 31.5% presenting with a single co-morbidity, 12.5% reporting two co-morbidities, and smaller fractions of participants accounting for 3 or more co-morbidities. Smoking status exhibited an association with symptom prevalence, as evidenced by the ascending mean number of symptoms: 4.32 ± 2.82 in smokers, 4.02 ± 1.86 in ex-smokers, and 3.90 ± 1.99 in non-smokers. However, due to the significantly limited representation of alcoholic and non-alcoholic individuals (285 vs. 4), as well as the disparity in vaccination status (269 vs. 20), statistical conclusions could not be drawn in these cases. Notably, participants who received a booster dose displayed a comparatively lower mean symptom count (3.85 ± 2.01), contrasting with those who did not receive the booster dose (4.53 ± 2.21). Conversely, upon examining the presence of specific medical conditions such as diabetes, hypertension, ischemic heart disease, renal disease, and asthma, no substantial disparities in the mean number of symptoms were identified. The exception to this trend was observed in the case of smoking, where a statistically significant difference emerged.

Moreover, an examination of demographic attributes and the spectrum of post-COVID-19 symptoms was conducted in relation to the stratification of COVID-19 infection severity, categorized as mild, moderate, and severe. The outcomes of these comparative analyses are presented in Table 3 and Table 4, respectively. Within the study cohort, a subset of participants demonstrated ongoing radiological alterations in their chest X-rays, accounting for 10.4% of the total. A notable proportion of individuals (20.5%) exhibited abnormal results in spirometry assessments. This spirometry analysis further delineated the presence of obstructive spirometry in 15.9% of cases and restrictive spirometry in 5.2% of cases. Moreover, an investigation into the relationship between symptomatology and specific clinical findings was undertaken. Comparing individuals with non-resolving radiological changes on chest X-rays, it was observed that those within this subgroup exhibited a slightly higher mean number of symptoms as opposed to those without such changes (4.36 ± 2.38 vs. 3.92 ± 2.01). Similarly, a parallel trend was discerned in relation to abnormal spirometry results, where individuals displaying abnormal spirometry patterns reported a higher mean number of symptoms in comparison to those with normal spirometry results (4.44 ± 2.26 vs. 3.85 ± 1.95).

**Table 3:** Comparison of different groups per the severity of COVID-19 Infection

Severity of COVID-19	N = 289 (%)	Mild	Moderate	Severe	Chi-square	p-value
Gender						
Male	108	71	23	14	8.253	.016
Female	181	146	20	15		
Age Groups						
Up to 40 years	58	53	4	1	40.161	.001
41 to 65 years	191	147	25	19		
66 to 80 years	34	15	10	9		
Over 80 years	6	2	4	0		
Ethnicity						
British	264	210	39	25	42.12	.006
Non-British	25	13	7	5		
Smoking Habits						
Smoker	28	22	3	3	11.76	.019
Ex-smoker	54	160	33	14		
Non-smoker	207	35	7	12		
Alcohol Habit						
Drinker	285	216	40	29	11.61	.003
Non-drinker	4	1	3	0		
Vaccination Status						
Vaccinated for COVID-19	269	205	37	27	4.621	.321
Non-vaccinated for COVID-19	20	12	6	2		
Booster dose availed	240	181	33	26	2.373	.667
Booster dose availed	49	36	10	3		
Comorbidities						
Hypertension	65	36	14	15	21.05	.001
Diabetes mellitus type 2	40	15	11	14	42.54	.001
Ischemic heart disease	20	6	3	11	49.11	.001
Chronic kidney disease	18	9	4	5	8.327	.016
Asthma	64	51	5	8	3.488	.175

**Table 4:** Comparison of post-COVID symptoms with the severity of COVID-19 Infection GI: Gastrointestinal

Symptoms	Frequency (N = 289)	Mild	Moderate	Severe	Chi-square	p-value
Fatigue	261	197	40	25	0.966	.617
Myalgia	100	72	21	8	4.638	.098
Brain fog	135	113	15	8	9.163	.010
Shortness of breath with exertion	225	166	37	29	1.943	.379
Anxiety	159	120	21	19	1.951	.377
Anosmia	57	46	6	6	1.182	.554
Hair loss	13	7	5	2	5.791	.055
GI disturbance	21	19	3	0	2.818	.244
Migraine	34	25	8	1	3.883	.144
Loss of appetite	29	20	5	4	0.736	.692
Loss of libido	3	2	1	0	1.027	.598
Persistent body aches	78	51	17	9	4.684	.096
Recurrent respiratory tract infections	25	16	6	3	2.084	.353

In assessing various relationships, the correlation between the severity of COVID-19 pneumonia and the subsequent development of long COVID-19 symptoms was scrutinized. The results of this investigation revealed a weak inverse correlation, although the absence of statistical significance (r = -0.011, p = 0.993) underscored the subtlety of this relationship. Subsequent scrutiny centered on the potential link between the number of comorbidities and the severity of the COVID-19 infection. Notably, a robust and statistically significant positive correlation was unearthed, indicating that a higher burden of comorbidities was closely correlated with elevated disease severity (r = 0.408, p = 0.001). This observation elucidates the role of underlying health conditions in influencing the course of the COVID-19 illness. Moreover, an examination was undertaken to explore any possible correlation between the number of comorbidities and the prevalence of post-COVID syndrome. However, the analysis yielded results that indicated an absence of a substantive correlation between these variables (r = -0.015, p = 0.797), revealing the nuanced nature of the relationship between comorbidities and post-recovery symptoms. Additionally, the inquiry extended to the examination of the relationship between BMI and the number of symptoms experienced by individuals. Remarkably, a notable finding emerged in this context, with a very strong positive correlation observed. This correlation (r = 0.911, p = 0.05) underscores the potential impact of BMI on the manifestation of symptoms in individuals affected by COVID-19.

Furthermore, the exploration delved into the interconnectedness of specific post-COVID symptoms, revealing intriguing correlations. Specifically, correlations between certain symptoms were identified, manifesting in their co-occurrence. Notably, fatigue exhibited a significant correlation with brain fog and shortness of breath with exertion (r = 0.255 and r = 0.205, respectively; p-value = 0.001). Similarly, myalgia demonstrated a positive correlation with body aches and loss of appetite (r = 0.388 and r = 0.262, respectively; p-value = 0.001). Also, a positive correlation was established between brain fog and migraine (r = 0.215, p-value = 0.001). Anosmia displayed correlations with migraine, loss of appetite, and loss of libido (r = 0.219, r = 0.293, and r = 0.204, respectively; p-value = 0.001). Notably, loss of libido exhibited robust correlations with hair loss, gastrointestinal issues, and loss of appetite (r = 0.454, r = 0.357, and r = 0.307, respectively; p-value = 0.001). Conversely, no other statistically significant correlations of substantial magnitude were discerned. Additionally, a significant negative correlation was detected between receiving a booster dose and the number of post-COVID symptoms, suggesting that the administration of a booster dose may potentially contribute to a reduction in the number of post-COVID symptoms (r = -0.123, p-value = 0.037).

A multinomial regression model was employed to explore the potential predictors of post-COVID-19 symptoms in different groups. It was found that the male gender is a predictor of hair loss, anosmia, and migraine, while the female gender is more associated with loss of appetite and loss of libido, as shown in Table 5. It was further revealed that smoking might be a potential factor for multiple post-COVID symptoms. Smoking seemed to be a predictor of fatigue, brain fog, anxiety, migraines, and persistent body aches, as shown in Table 6.

**Table 5:** Multinomial regression analysis of variables for males vs. females

Symptoms	B	OR	p-value	95% CI	
				Lower	Upper
Fatigue	.101	1.10	.820	.462	2.67
Hair loss	1.35	3.85	.103	.762	19.5
Anosmia	.385	1.46	.289	.721	2.99
Myalgia	.236	1.26	.444	.692	2.31
Brain fog	.601	1.82	.029	1.06	3.12
Anxiety	.513	1.67	.052	.995	2.80
Migraine	1.30	3.69	.029	1.14	11.89
Loss of appetite	-.605	0.54	.274	.184	1.61
Loss of libido	-4.12	0.15	.001	.001	.436

**Table 6:** Multinomial regression analysis of variables for smokers vs. non-smokers

Symptoms	B	OR	p-value	95% CI	
				Lower	Upper
Fatigue	.267	1.30	.718	.307	5.55
Brain fog	.239	1.27	.606	.512	3.14
Anxiety	.672	1.95	.150	.784	4.89
Migraine	.594	1.81	.452	.385	8.52
Body aches	.288	1.33	.615	.434	4.09

## Discussion

The COVID-19 pandemic has been the most devastating one in recent history, affecting millions of people worldwide. Many individuals who contracted the viral disease and subsequently recovered still experienced persistent symptoms that could not be attributed to any other medical condition. This led to the understanding of a new phenomenon known as post-COVID-19 syndrome, or long COVID [16]. Our study aimed to investigate the potential correlation between the symptoms of long COVID syndrome and patient characteristics, including age, sex, smoking habits, vaccination status, the severity of the acute infection, and comorbidities. Our study yielded intriguing insights; however, many points still require further research.

The most common symptom reported by patients was fatigue, followed by dyspnea on physical exertion, anxiety, brain fog, and myalgia. Other symptoms, such as anosmia, migraines, loss of appetite, recurrent respiratory infections, gastrointestinal upset, hair loss, and loss of libido, were also reported in a significant percentage. This symptom profile is similar to that demonstrated by a cross-sectional study conducted in Germany by Lemhöfer et al. in 2021 [17]. However, Lemhöfer et al. did not include patients who had severe COVID-19 requiring hospitalization in their study. Fatigue was also the most commonly reported symptom in a study by Sudre et al., in which data were collected from a mobile application launched in response to the COVID-19 pandemic [18]. Interestingly, coughing, which is typically associated with other post-viral syndromes, did not emerge as a significant symptom in our study. This finding is consistent with other studies, in which cough was not among the most frequent symptoms of long COVID [19].

The present study did not find any statistically significant association between the severity of COVID-19 pneumonia and the incidence of long COVID syndrome. This finding is consistent with other studies as well. Townsend et al. conducted a cross-sectional study in 2021 to assess symptoms of fatigue and dyspnea in COVID-19 patients who came for follow-up at least six weeks after their acute illness [20]. The participants were divided into three groups based on disease severity, but no correlation was found between the incidence of long COVID symptoms and the initial severity of the disease. Van den Borst et al. conducted a prospective observational study in which they utilized the short form-36 (SF-36) and Nijmegen clinical screening instrument (NCSI) to evaluate symptoms associated with long COVID [21]. In their study, patients with mild disease severity had significantly lower health status scores compared to those with moderate and severe disease.

The number of comorbidities was found to have no significant association with long COVID. Comorbidities such as diabetes, hypertension, ischemic heart disease, renal disease, and asthma were assessed, and no correlation was observed between the number of long COVID-19 symptoms and these comorbidities. A case-control study conducted by Fernández-de-Las-Peñas et al. reports similar results, demonstrating no difference in the number of long COVID symptoms between individuals with diabetes and those without diabetes [22]. Munblit et al., in a longitudinal cohort study evaluating 2,649 patients, did not find any positive association between asthma and long COVID [23]. Carvalho-Schneider et al. also reported that the number of comorbidities was not a predictor of long COVID syndrome [24]. However, in contrast to the findings of our study, Chen et al. have shown in their meta-analysis that symptoms of long COVID are positively correlated with a previous history of comorbidities, such as asthma [25]. Sudre et al. also reported that asthma was the only pre-existing condition positively associated with long COVID-19 [18].

Our research shows that obesity and female sex are strong predictors of long COVID symptoms. Our study suggests that male sex is a significant predictor of hair loss, anosmia, and migraine. On the other hand, the female gender is more closely associated with a decrease in appetite and libido. This finding aligns with other studies in the literature.

It is worth noting that males have been associated with higher morbidity and mortality rates in acute COVID-19 infections [26]. However, the female sex is associated with an increased burden of symptoms in long COVID. Notarte et al. conducted a systematic review and meta-analysis, which demonstrated that obesity and female sex are risk factors for long COVID [27]. Several other studies have also reported an association between long COVID and the female sex. Peghin et al. conducted a bidirectional cohort study with 599 participants [28]. The study found that the female sex is a significant predictor of long COVID. Desgranges et al. also show that obesity and the female sex are significant risk factors for long COVID [29]. Gebhard et al. examine the sociocultural factors that may contribute to the higher prevalence of long COVID in women [30]. Differences in immunological responses between males and females could also be used to explain the sex-based disparities observed in both acute COVID-19 infection and long COVID syndrome [31]. However, the specific mechanism behind this difference is still under research.

It was also observed that smoking could contribute to various post-COVID-19 symptoms. Smoking appeared to be a predictor of fatigue, brain fog, anxiety, migraines, and persistent body aches. We also found no evidence of a correlation between age and long COVID symptoms. It is worth noting that old age has been associated with poor outcomes in COVID-19 pneumonia. However, the role of increasing age in long COVID is still uncertain. While some studies have shown that old age is associated with long COVID, e.g., Thompson et al. [32], others, like a meta-analysis by Maglietta et al. [33], do not demonstrate any such association. Our findings will contribute to the existing body of evidence and published literature.

In this study, a significant negative correlation was found between administering a booster dose and the occurrence of post-COVID-19 symptoms. This suggests that receiving a booster dose may potentially reduce the number of symptoms associated with long COVID. A systematic review and meta-analysis by Marra et al., including six studies, evaluated data from 251,123 individuals [34]. Their study concluded that vaccination was effective in preventing long COVID in both individuals who received the vaccine before contracting COVID-19 and those who received it afterwards. A cohort study by Taquet et al. concluded that two doses of the COVID-19 vaccine had a protective effect against long COVID compared to a single vaccine dose [35].

In the present study, the average number of long COVID symptoms was higher in patients who had persistent radiological changes on chest X-rays and abnormal spirometry results during follow-up. This may indicate respiratory pathology as a potential cause for certain features of long COVID. A prospective cohort study conducted by Zhao et al. reported that 25.45% of COVID-19 patients exhibited abnormal lung function on spirometry, while 70.9% showed CT chest abnormalities three months after being discharged from the hospital [36]. However, since long COVID-19 may involve multiple systems, relying solely on respiratory parameters may not be sufficient to fully explain the wide range of long COVID symptoms.

## Conclusions

The most frequently reported symptoms among patients with long COVID include fatigue, dyspnea on physical exertion, anxiety, brain fog, and myalgia. The severity of the initial COVID-19 infection, as determined by factors such as hospitalization, did not show a statistically significant association with the number of long COVID symptoms. Several factors, including obesity, female sex, and a history of smoking, were found to be strong predictors of long COVID symptoms. Administering a booster dose was found to have a negative correlation with the occurrence of long COVID symptoms. This suggests that receiving a booster dose may decrease the symptoms of long COVID. Patients who had persistent radiological changes on chest X-rays and abnormal spirometry results during follow-up exhibited a higher average number of long COVID symptoms. This suggests a potential connection between respiratory pathology and the specific characteristics of long COVID.
